# Continuing to look in the mirror: A review of neuroscientific evidence for the broken mirror hypothesis, EP-M model and STORM model of autism spectrum conditions

**DOI:** 10.1177/1362361320936945

**Published:** 2020-07-15

**Authors:** Luke Yates, Hannah Hobson

**Affiliations:** University of York, UK

**Keywords:** Autism spectrum condition, imitation, mirror neurons, top-down control

## Abstract

The mirror neuron system has been argued to be a key brain system responsible for action understanding and imitation. Subsequently, mirror neuron system dysfunction has therefore been proposed to explain the social deficits manifested within autism spectrum condition, an approach referred to as the broken mirror hypothesis. Despite excitement surrounding this hypothesis, extensive research has produced insufficient evidence to support the broken mirror hypothesis in its pure form, and instead two alternative models have been formulated: EP-M model and the social top-down response modulation (STORM) model. All models suggest some dysfunction regarding the mirror neuron system in autism spectrum condition, be that within the mirror neuron system itself or systems that regulate the mirror neuron system. This literature review compares these three models in regard to recent neuroscientific investigations. This review concludes that there is insufficient support for the broken mirror hypothesis, but converging evidence supports an integrated EP-M and STORM model.

**Lay abstract:**

The mirror neuron system has been argued to be a key brain system responsible for understanding the actions of others and for imitation. It has therefore been proposed that problems within this system could explain the social difficulties experienced by people with autism spectrum condition. This idea is referred to as the broken mirror hypothesis. However, research has produced insufficient evidence to support the broken mirror hypothesis in its original form. Therefore, two other models have been suggested: EP-M model and the social top-down response modulation (STORM) model. All models suggest something is different regarding the mirror neuron system in autism spectrum condition: either within the mirror neuron system itself or within the systems that control the activity of the mirror neuron system. This literature review compares these three models in regard to recent neuroscientific investigations. This review concludes that there is insufficient support for both the broken mirror hypothesis, but converging evidence supports an integrated EP-M and STORM model.

The discovery of mirror neurons, in macaque monkeys, was considered by some to be a major breakthrough into unravelling the neural basis of action understanding, imitation and also Autism spectrum condition (hereafter ASC/autism) (di Pellegrino et al., 1992). The mirror neuron system (MNS), comprising mainly the inferior frontal gyrus (IFG) and inferior parietal lobe (IPL), responds when actions are performed (e.g. grasping a cup to drink) and when the same action is observed (seeing another person grasp a cup). This visual-motor property links self-generated actions with the actions of others, theoretically allowing an individual to understand observed actions in terms of their own movements (Buccino et al., 2004). Since action understanding has been argued to be impaired in ASC (Boria et al., 2009; Cattaneo et al., 2007; Rizzolatti et al., 2009; Vivanti et al., 2011; see also Hamilton et al., 2007), an MNS dysfunction was suggested to explain the condition; this is essentially the broken mirror hypothesis (BMH) (Ramachandran & Oberman, 2006).

However, the function and even the very existence of the human MNS remain controversial. There have been methodological issues in MNS-related research, including the use of unsatisfactory controls and confounding variables, such as the effect of attention on specific electroencephalographic responses (Hobson & Bishop, 2016, 2017), as well as the notion that data is predominantly correlational rather than causational. Counter-arguments have also highlighted that data from primates cannot be used to explain the abilities that primates themselves lack, such as language (see Hickok, 2009). Indeed, human and animal studies have discrepant methodologies; while studies on macaques have been able to use single-cell recordings to demonstrate the response patterns of cells in the sensorimotor cortex, such a technique is rarely able to be applied in research studies of the human MNS, which has had to rely on more indirect measures of the MNS, including functional magnetic resonance imagining (fMRI) and EEG. Importantly for the present review, some studies have also failed to replicate any MNS abnormalities in ASC (Fan et al., 2010; Mathewson et al., 2012). To reconcile the inconsistencies within the literature, Hamilton conducted two reviews into the BMH (Hamilton, 2008, 2013). Both reviews concluded that there is a lack of evidence for the BMH and instead proposed two alternate models: the EP-M model (Hamilton, 2008) and the social top-down response modulation (STORM) model (Hamilton, 2013; Wang & Hamilton, 2012). All three models are summarised in Figure 1.

**Figure 1.**
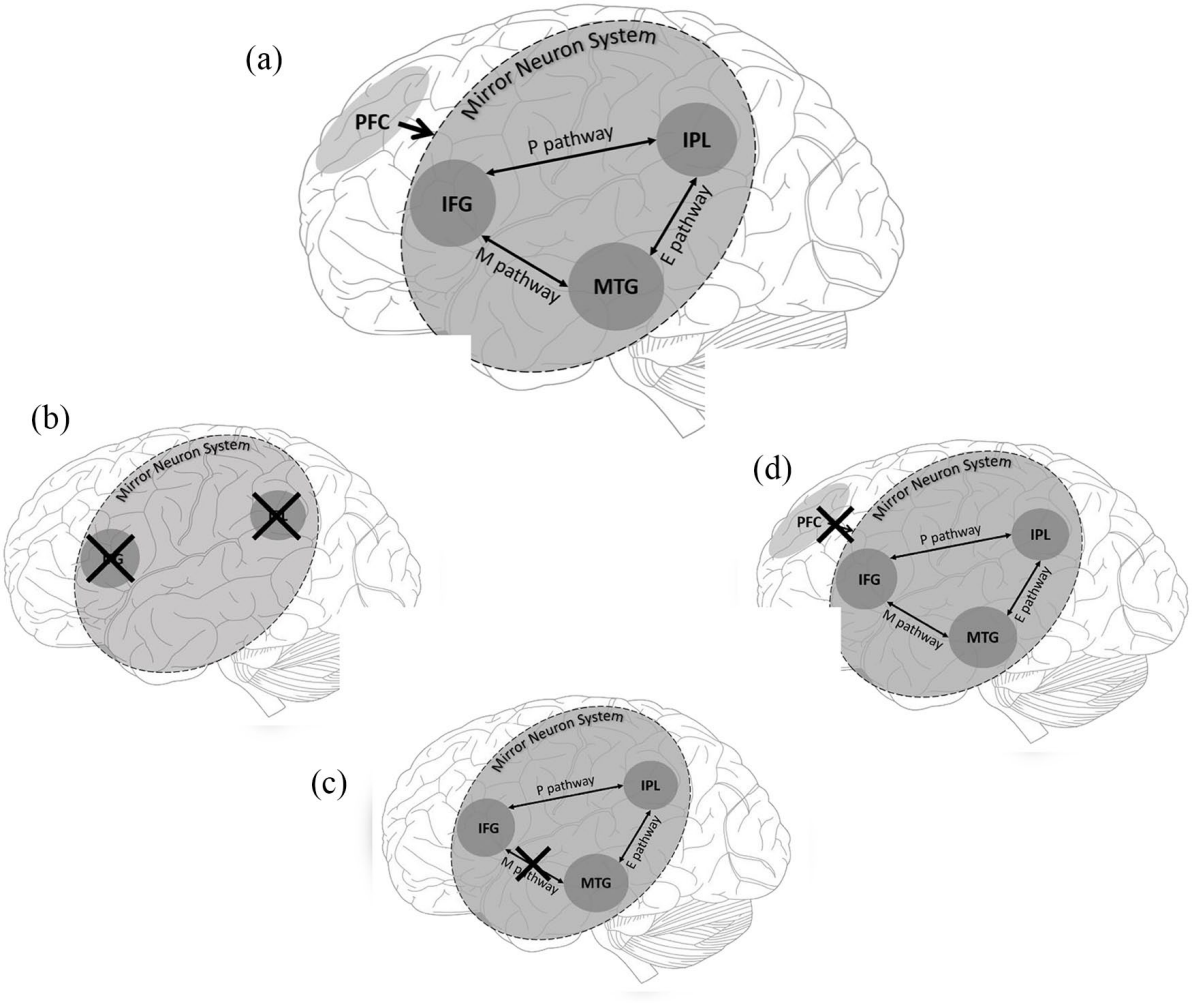
Nodes of the mirror neuron system (MNS) and areas of top-down control as included in the social top-down response modulation (STORM) model, EP-M model and broken mirror hypothesis (BMH) of autism spectrum condition. The crosses indicate suggested locations of abnormality/dysfunction proposed by different models. (a) This includes all regions and pathways across the three models. (b) This represents global dysfunction of the MNS stated by the BMH. (c) This demonstrates dysfunction to the Mimicry Pathway (M) but no dysfunction to the planning (P) or emulation (E) pathway of the MNS, proposed by the EP-M model. (d) This represents how top-down dysfunction could disrupt appropriate MNS functioning as suggested by the STORM model. PFC: prefrontal cortex; IFG: inferior frontal gyrus; IPL: inferior parietal lobe; MTG: middle temporal gyrus.

## The EP-M model and STORM model

A main tenet of the EP-M model (Figure 1(b)) is that the pattern of behavioural difficulties and strengths in autism, particularly in regard to imitation abilities, does not support a global difficulty in the MNS. Instead, imitation behaviour is served by two routes of three nodes, the IFG, the IPL and the middle temporal gyrus (MTG), and three pathways between them. Processing of all actions begins in the MTG, which extracts the visual kinematic features (e.g. the motion of a hand) of an observed action. The remaining route is then based on whether the action is goal-directed or not. For goal-directed actions, information is then sent from the MTG via the emulation pathway (E-route) to the IPL. The IPL then processes the action’s abstract goal (e.g. grasping a cup to drink). This goal information is then passed on to the IFG, via the planning (P) pathway, where the motor features for action execution are formulated. If the action to be imitated is not goal-directed, this is served by the mimicry route (M-route), a direct route between the MTG and IFG, which provides a direct connection between the visual features of observed actions and motor representations. This allows for automatic imitation of observed action sequences. It is this mimicry route that is claimed to be impaired in ASC, according to the EP-M model.

The STORM model (Figure 1(d)) proposes that ASC symptoms stem from abnormalities within the top-down regulation of the MNS, rather than within the MNS itself. In NT (neurotypical) individuals, the MNS processes the visual-motor properties of executed/observed actions, while the medial prefrontal cortex (mPFC) imposes the social significance of those actions onto the MNS. For example, observing someone raising their arm will be processed differently depending on whether they are in a lecture or at a supermarket. This top-down modulation is argued to be reduced in ASC, resulting in an impaired ability to utilise the social relevance of observed actions but a regular ability to imitate actions (via visual-motor integration).

These models are not mutually exclusive: Hamilton (2008) suggested that the M-route dysfunction proposed in the EP-M model could stem from atypicalities in top-down modulation. However, both models contrast with the BMH which claims that a global deficit in the MNS explains ASC symptomology.

## Aims of the current review

Given continued neuroscientific research into the MNS in ASC, a review and comparison of these two models is timely. The present literature review aims to critically assess studies published since Hamilton’s reviews, to capture the extent to which each model is supported by recent neuroscientific findings. Behavioural evidence (i.e. imitation deficits in autism) has been reviewed elsewhere, demonstrating robust deficits in imitation in autistic individuals (Edwards, 2014). Interestingly, this deficit was argued to be specific to tasks which required participants to reproduce the precise movements of the action to achieve a goal, and ASC participants showed no difference to NT participants when they could use their own method of execution to imitate the goal of a task (emulation). Such a review is in keeping with the EP-M model.

However, all three models – the BMH, EP-M and STORM – are inherently neuroscientific models that make claims regarding the role of specific brain networks and regions. Therefore, our review shall focus on neuroscientific methodologies (i.e. methods of inquiry that draw upon measures of brain activity or structure), to complement reviews of behavioural work. Within this definition of ‘neuroscientific’ also falls neurofeedback training (NFT) and post-mortem data, neither of which were included in Hamilton’s reviews.

## Predictions from respective theories

Each model yields specific predictions for neuroscientific data. If the BMH is valid, then participants with ASC should show a global deficit in the MNS, leading to a disadvantage in tasks reliant upon this system: as long as the task utilises mirroring mechanisms, there should be a significant difference between ASC participants and NT participants on measures of mirror neuron activity. Whether this difference is hyperactivity or hypoactivity in relation to NT controls is not specified, and both have been argued to support the theory (Dapretto et al., 2006; Martineau et al., 2010; Williams et al., 2006).

The EP-M model would predict that ASC individuals are only disadvantaged on tasks in which participants process non-goal-directed actions, as these depend on the M-route. Tasks in which stimuli have an end goal, and therefore utilise the EP pathway, should produce no significant differences between ASC and NT participants on measures of mirror neuron activity.

In contrast to both of the preceding models, the STORM model would predict that differences between NT and ASC groups are not due to deficits within the MNS itself but within its connections to and regulation by frontal regions of the brain. This would suggest that under circumstances that did not require top-down regulation of the MNS, ASC and NT participants should appear the same. However, ASC individuals would be disadvantaged if the task had social relevance or relied upon the executive control of the MNS.

## Method

A literature search, incorporating Ovid, Web of Science and Google Scholar, was conducted using the following search phases: ‘Autism’ OR ‘ASD’ OR ‘ASC’ OR ‘Autistic’ AND ‘Mirror neuron system’ OR ‘MNS’ OR ‘Broken Mirror Hypothesis’. Initial keyword searches produced 7088 papers (Ovid = 184, Google scholar = 38, Web of Science = 6866). Papers were selected based on the following criteria: (1) they must refer to both ASC and the MNS; (2) they must focus on the MNS rather than the whole action imitation network; (3) they must not have been incorporated in Hamilton’s 2008 or 2013 review; (4) they must not be a systematic review and (5) they must use quantitative neurological measures rather than behavioural or qualitative measures. Screening abstracts, titles and methods sections in reference to this inclusion criteria revealed 17 papers of relevance. Papers on NFT published prior to Hamilton’s reviews were included as these studies were not incorporated in these previous reviews. These 17 papers, dated from 2012 to 2018, are briefly summarised in Table 1.

**Table 1.** Summary of studies included in review, by methodology.

EEG studies				
Paper	ASC sample characteristics	Control sample characteristics	Task/procedure	Results
Oberman et al. (2013)	*N* = 66, Age range = 6–17 y NFemales = 0 IQ not specified Diagnoses: ASD, autistic disorder, Asperger’s disorder, PDD-NOS	*N* = 51 Age range = 6–17 y NFemales = 0 IQ not specified	Pooled data from 4 studies using a biological movement observation task. Stimuli included videos of a stranger’s hand opening and closing (observation condition). Baseline condition included either rest or non-biological motion.	ASC participants showed significantly less mu suppression than NT participants in the observation condition only. Equally strong correlation between age and mu suppression in both ASC and NT groups.
Ruysschaert et al. (2014)	*N* = 18 Age range = 2.3–5.0 y NFemales = 4 IQ not specified diagnoses: ASD, PDD-NOS	*N* = 19 Age range = 2.1–4.9 y NFemales = 11 IQ not specified	Observed goal-directed hand movements: watched the experimenter place a toy inside a box in a wave movement then were tasked on imitating this action. Also observed hand movements without the object. Baseline condition watching a dangling object swing back and forth.	No group differences in mu suppression during observation or execution of goal-directed hand movements, nor in non-object-directed action observation. No correlation between mu suppression and age, imitation ability or social communication score.
Bernier et al. (2013)	*N* = 19 Age range not specified, Mean age = 6.9 y NFemales = 1 Mean FSIQ = 118.3 Diagnoses: ASD	*N* = 19 Age range not specified, mean age = 6.4 y NFemales = 2 Mean FSIQ = 95.5	Observed/executed identical goal-directed hand actions (grasping a wooden block). Baseline was a rest condition. Also completed the mature imitation task.	Mu suppression related to facial imitation ability, not hand imitation. No group differences in average mu suppression. Subset of participants in both groups showed reduced mu suppression and had lower imitation scores. No relation between autism-related communication impairment and mu rhythm attenuation.
Dumas et al. (2014)	*N* = 10, Age range = 21–41 y, NFemales = 3 IQ not specified diagnoses: ASD	*N* = 30 Age range = 20–39 yNFemales = 16 IQ not specified	Observed meaningless hand actions. Could then imitate these hand actions or produce their own hand actions. Also included condition where participants were required to imitate the video they saw. Baseline included resting condition.	ASC participants showed reduced mu suppression during action observation, when analysing the whole mu band (8–13 Hz), over the central electrodes. When the mu band split, there were no group differences in lower band (8–10 Hz) suppression. Higher upper band (10–12/13 Hz) suppression in NT participants during observation. NT participants showed greater suppression of upper band other occipital-parietal electrodes while the ASC group showed greater upper band suppression over frontal electrodes.
TMS studies				
Paper	ASC sample characteristics	Control sample characteristics	Task/procedure	Results
Enticott et al. (2013)	*N* = 32 Age range not specified, mean age 24.8 y NFemales = 8 Mean FSIQ = 104.63 Diagnoses: ASD, Asperger’s Disorder	*N* = 32 Age range not specified, mean age = 25.5 y NFemales = 9 Mean FSIQ = 112.13	Watched videos of 5 types of hand actions: static hands, individual approach (left and right hand from the same person clasping together), interacting approach (left and right hands from different people clasping together), individual removal (left and right hand from same individual releasing a clasp), interacting removal (left hand moves from the left of screen to touch another person’s right hand, but the other person retracts to avoid the touch). Baseline was static hand condition.	No main effect of group for all types of movements.
TMS + EEG studies				
Paper	ASC sample characteristics	Control sample characteristics	Task/procedure	Results
Cole et al. (2018)	*N* = 13 Mean age = 28.3 y NFemales = 4 Mean VIQ = 111.62 Diagnoses: Asperger syndrome, ASD	Low AQ Control: *N* = 15 Mean age = 23.40 y NFemales = 7 Mean VIQ = 109.67 High AQ Control: *N* = 15 Mean age = 24.13 y NFemales = 8 Mean VIQ = 113.00	Mentalising task: State whether actor was demonstrating a clumsy or spiteful action (failing to post a poker chip through a slot). Non-mentalising task: State whether actor accomplished a successful or unsuccessful action (posting a poker chip through a slot). Baseline included resting state.	Significant group effects in right mu suppression at 8–10 Hz during both tasks. However, for the effects during the non-mentalising task, this effect was driven by differences between the high–low AQ control groups, not between NT and ASC groups. Level of autistic traits predicted right mu suppression at 8–10 Hz only during the mentalising task. Level of left mu suppression predicted mentalising performance across all participants. No group differences at 10–12 Hz. No group difference in MEPs during mentalising or non-mentalising task.
fMRI				
Paper	ASC sample characteristics	Control sample characteristics	Task/procedure	Results
Fishman et al. (2014)	*N* = 25 Age range = 11.8–17.7 y NFemales = 3 Mean FSIQ = 113 Diagnoses: ASD	*N* = 25 Age range = 12.1–16.8 y NFemales = 5 Mean FSIQ = 108	Resting	Increased connectivity in MNS in ASC participants with the greatest autistic symptoms compared to NT participants. No difference between total ASC group and NT group in connectivity in MNS. ASC participants demonstrated over-connectivity between mPFC, SPL and MTG, and under-connectivity between bilateral TPJ STG and the precuneus. Greater connectivity between theory of mind system and MNS correlated with decreased social impairments.
Libero et al. (2014)	*N* = 21, Age range = 17–40 y NFemales = 4 Mean FSIQ = 116.3 Diagnoses: ASD	*N* = 22 Age range = 19–36 y NFemales = 5 Mean FSIQ = 117.5	Viewed static images of actions using objects. Intention task: State whether the intention of the action was ordinary or unusual. Means task: Decide whether the task was carried out was in an ordinary or unusual way	Determining the intention of the action activated STG, MTG and precuneus in both groups. Determining the means of how the goal was accomplished activated the IPL and occipital regions. ASC group demonstrated reduced precuneus activity when evaluating the intention, but increased left inferior frontal gyrus activity when examining the means. Increased activity in the pre- and post-central gyrus, right IFG and left IPL was found in the ASC group when viewing unusual actions. Weaker frontal–temporal connections in the ASC group when inferring intentions. The activity of the PCC positively correlated with empathising ability and negatively with autism symptom severity. NT participants were more accurate in both tasks than ASC participants, but there was no difference in reaction time.
Wadsworth et al. (2018)	*N* = 13 Age range = 8–17 y NFemales = 2 Mean FSIQ = 109 Diagnoses: ASD	*N* = 15 Age range = 9–15 y NFemales = 4 Mean FSIQ = 102	Participants shown images of everyday tasks being performed with hands missing. Participants had to choose which of the 3 displayed hand positions fitted the picture of the task. Included transitive and intransitive actions.	No group differences in performance accuracy. IFG activated in both groups. ASC group demonstrated increased activity in right MTG and vPM for actions which lacked an object. Autism severity correlated with activity right vPM, right MTG and lateral superior parietal lobe, for actions which lacked an object.
NIRS studies				
Paper	ASC sample characteristics	Control sample characteristics	Task/procedure	Results
Mori et al. (2015)	*N* = 10 Age range = 9–14 y NFemales = 0 Mean FSIQ = 82.5 Diagnoses: Autistic disorder	*N* = 10 Age range = 9–14 y NFemales = 0 IQ not specified	Imitate an emotional face (happy, sadness, surprise, anger, disgust and fear), presented on screen. ASC group also underwent a 30-min training session on imitating emotional faces	Lower concentration of Oxyhaemoglobin in the IFG in the ASC group when imitating emotional faces compared to controls. ASC participants demonstrated elevated oxyhaemoglobin levels post training compared to pre-training.
DTI/DSI studies				
Paper	ASC sample characteristics	Control sample characteristics	Task/procedure	Results
Schaer et al. (2013)	*N* = 11 Age range = 9.3–17.4 y NFemales = 3 Mean FSIQ = 79.4 Diagnoses included: ASD	*N* = 11 Age range = 8.7–16.8 y NFemales = 3 Mean FSIQ = 110.5	Resting	No group differences in cortical volume or MNS connectivity. Reduced gyrification in the ASC group in right IPL, precentral gyrus, IFG and medial parieto-occipital region.
Chien et al. (2015)	*N* = 36 Age range = 15–26 y NFemales = 2 Mean FSIQ = 101 Diagnoses included: ASD	*N* = 30 Age range = 14–25 y NFemales = 3 Mean FSIQ = 107	Resting	No group differences in cortical thickness in IFG or SMG. No differences in tract integrity, including in tracts connecting IFG and SMG. The NT group showed structural covariance (the cortical thickness of the regions of interest correlated), but this was not true for the ASC group. Social communication problems correlated with the integrity of the right IFG–SMG tract.
Fründt et al. (2018)	*N* = 15 Age range = 20–49 y NFemales = 5 Mean FSIQ = 112.5 Diagnoses included: ASD	*N* = 13 Age range = 24–44 y NFemales = 8 Mean FSIQ = 105.9	Resting	Intact frontal-parietal MNS pathway found in both ASC and NT groups. No evidence for fronto-temporal MNS pathway in either group. The ASC group showed a significant negative correlation between empathy quotient scores and fractional anisotropy values of the right frontal-parietal tract.
Post-mortem analysis studies				
Paper	ASC sample characteristics	Control sample characteristics	Task/procedure	Results
Jacot-Descombes et al. (2012)	*N* = 8, Age range = 4–66 y NFemales = 2 IQ not specified Diagnoses included: ASD	*N* = 2 Age range = 4–45 y NFemales = 1 IQ not specified	Post-mortem examination of inferior frontal gyrus (Brodmann areas 44 and 45).	No group differences in pyramidal volume or density of neurons in each layer. Significantly smaller neurons found in ASC brains (in layer III).
Neurofeedback training + fMRI studies				
Paper	ASC sample characteristics	Control sample characteristics	Task/procedure	Results
Datko et al. (2018)	*N* = 10 Age range = 9.26–18.3 y NFemales = 3 Mean FSIQ = 101.1 Diagnoses included: ASC	*N* = 7 Age range = 8.64–16.7 y NFemales = 2 Mean FSIQ = 115.6	Observed a hand and then imitated hand pressing a button. Played a game (making a car drive round the screen) or watched a film during NFT.	No group difference in accuracy of imitation before or after NFT. ASC group improved social responsiveness after NFT. ASC group demonstrated higher activity in right inferior parietal lobe (IPL) after NFT than before NFT. NT showed lower activity in precentral gyrus, IFG, left IPL, SMG and occipital areas after NFT.
Neurofeedback training only studies				
Paper	ASC sample characteristics	Control sample characteristics	Task/procedure	Results
Pineda et al. (2008)	Experimental group: *N* = 9 Age range not specified, mean age = 9.4 y NFemales = 3 FSIQ > 80 Diagnoses included: ASD	Placebo group: *N* = 10 Age range not specified, mean age = 10.1 y NFemales = 0 FSIQ > 80 Diagnoses included: ASD	Watched silent movies to measure mu suppression outside of training: ball bouncing, hand moving to take imaginary crayon; hand takes actual crayon; people passing a ball around; biological motion. During NFT: Played video game while being encouraged to keep a left-hand bar (representing mu suppression) above threshold and a right-hand bar (representing muscle activity) below threshold.	The experimental group showed more mu suppression than placebo group when watching the hand, crayon, social and happy face videos. Larger improvement in ADHD scores in experimental group than placebo.
Pineda et al. (2014)	*N* = 10 Age range = 7–17 y NFemale = 0 IQ not specified Diagnoses included: ASC	*N* = 10 Age range = 8–17 y NFemale = 0 IQ not specified	Watched three 15-min video clips or 1-h-long video. If theta bands exceeded threshold the video would pause. To resume playing the video, mu-rhythms would have to go above a predetermined threshold for 1 s.	The ASC group showed greater control over mu-band power after NFT than NT group. ASC group increased in social responsiveness and reduced autistic severity scores. NT reduced in social responsiveness (beyond the ASC diagnosis threshold) and increased in autism severity scores (still below threshold).

EEG: electroencephalography; ASC: autism spectrum condition; IQ: intelligence quotient; ASD: autism spectrum disorder; PDD-NOS: pervasive developmental disorder – not otherwise specified; NT: neurotypical; FSIQ: full-scale intelligence quotient; TMS: transcranial magnetic stimulation; VIQ: verbal intelligence quotient; AQ: autism spectrum quotient; fMRI: functional magnetic resonance imagining; MNS: mirror neuron system; NT: neurotypical; mPFC: medial prefrontal cortex; SPL: superior parietal lobule; MTG: medial temporal gyrus; TPJ: temporoparietal junction; STG: superior temporal gyrus; IFG: inferior frontal gyrus; IPL: inferior parietal lobule; vPM: ventral premotor cortex; NIRS: near-infrared spectroscopy; DTI: diffusion tensor imaging; DSI: diffusion spectrum imaging; SMG: supramarginal gyrus; NFT: neurofeedback training; ADHD: attention-deficit hyperactivity disorder; PCC: posterior cingulate cortex;.

## Results

### Evidence from electroencephalography (EEG) studies

In EEG studies of the MNS, a common index of MNS activity is mu suppression, a characteristic reduction in alpha activity (8–13 Hz) from electrodes over the sensorimotor strip (i.e. typically sensors C1, C2, C3) during action observation or execution (Braadbaart et al., 2013). If indeed autistic participants have a dysfunctional MNS, as proposed by the BMH, ASC participants should show a lack of mu suppression or reduced mu suppression compared to controls during action observation. In comparison, the EP-M model would suggest that this lack of mu suppression should only be seen for conditions in which action stimuli or tasks lack a specific goal. The STORM model would only predict less mu suppression in ASC participants if input from regions such as the PFC was required to complete the task (i.e. to compute the social relevance of a situation within the task).

Comparing data from six studies, Hamilton (2013) found no clear evidence to support the BMH and advocated for the STORM model. For the current review, five additional studies meeting the search criteria were found, which compared mu suppression in the ASC and NT groups. In each study, EEG recordings were extracted, while the participant watched a video or a live demonstration of a biological action (e.g. a stranger opening and closing their hand, watching the experimenter place a toy in a box). Baseline conditions included either a rest condition or watching a non-biological action (i.e. a ball moving). Some researchers have proposed utilising the mu rhythm as a target for neurofeedback therapy: these studies are discussed in a subsequent section.

Oberman et al. (2013) attempted to investigate differences in developmental changes in the MNS in NT versus ASC groups, by pooling data from four previous mu suppression studies and examining the correlation between mu suppression and age. Age was associated with mu suppression, with stronger mu suppression to observing action stimuli with increasing age. The correlations did not differ between the ASC and NT groups, suggesting that the MNS develops along a similar trajectory in ASC and NT children. This pooled analysis did show a significant difference between NT and ASC groups in their mean mu suppression when observing action stimuli, which would support the BMH. However, arguably the EP-M model is also supported as the action observation condition for three out of the four studies’ data was exclusively from conditions that were actions without objects or goals (opening and closing a hand). These are actions that would be theoretically reliant on the M-pathway. The fourth dataset would have included both object-based and non-objected-based actions, and contributed only 8/66 ASC datasets. The results would thus be in-keeping with a dysfunction in the M-pathway.

Ruysschaert et al. (2014) investigated mu suppression in young children, using goal-based and non-goal-based stimuli: they report no significant group effects, nor any group by condition interactions. Thus, no deficits in mu suppression were seen for either kind of action. These results fit neither with the BMH nor the EP-M, but could be interpreted according to the STORM framework, which would predict abnormalities in the MNS only under task conditions which demand social top-down regulation.

Bernier et al. (2013) investigated the association between mu suppression and behavioural imitation abilities, in children with ASC and NT controls. In addition to the EEG procedure, in which participants observed and executed the action of grasping a wooden block, each child also completed a behavioural imitation task, which included tests of manual and facial imitation abilities. ASC and NT participants showed comparable mu suppression and imitative ability. These results are problematic for the BMH, although Bernier et al. (2013) noted that there was a sub-group of participants who demonstrated a lack of mu suppression and also poor imitation, derived from both the ASC and NT samples; the authors suggest that behaviours such as imitation, rather than autism per se, may be associated with MNS atypicalities. Mu suppression correlated with facial imitation abilities only. Arguably, facial imitation may have a greater reliance on MNS systems, given that one’s own face cannot be seen (as opposed to one’s hands), and thus action–observation matching mechanisms may be a more central process for this type of imitation. Facial imitation was also noted in Hamilton (2008) as being particularly reliant on the M-route, and thus these results also support the EP-M model. Furthermore, the EEG action observation condition involved actions where a hand grapes an object, an action that would be served by the EP route. As this route is intact in ASC according to Hamilton (2008), the lack of group differences supports the EP-M model. Finally, given there was no social top-down regulation required in this study’s procedure, the lack of group differences in mu suppression is also in keeping with the STORM model.

So far, the discussed investigations have focused on the 8–13 Hz rhythm as a single band width and focused dominantly on the central electrodes (positioned above the sensorimotor strip). Dumas et al. (2014) extended their EEG analysis to the whole scalp and split the mu frequency band into two sub-bands. Participants observed videos of meaningless hand gestures, imitated these hand gestures and produced their own. It was demonstrated that, when observing hand actions, adults with ASC show reduced mu suppression in the upper sub-band (10–12/13 Hz), yet demonstrated no differences to NT participants in the lower sub-band (8–10 Hz). These sub-bands have been attributed to different brain areas; the lower band has been attributed to the MNS, while the upper band may arise from executive control areas (Frenkel-Toledo et al., 2014). Therefore, these results are supportive of differences in top-down modulation. Furthermore, mu suppression differences arose in parietal–occipital regions (caudal to the regions typically implicated in mu suppression studies that only consider electrode sites over the sensorimotor strip) and frontal regions of the brain, rather than the sensorimotor regions. This suggests that differences between groups lie outside the typical MNS regions.

Finally, Cole et al. (2018) utilised both EEG and transcranial magnetic stimulation (TMS) measures of the MNS in their investigation (TMS findings are considered in the section below). Their conditions included a mentalising task (stating whether an actor was spiteful or clumsy when failing to post a poker chip through a slot) and a non-mentalising task (stating whether an actor was successful or unsuccessful at posting a poker chip through a slot). They also used the approach of Dumas et al. (2014), splitting their analyses into upper and lower mu bands. There were no group effects on the upper mu bands. For the lower mu band, there were significant differences between ASC participants and NT participants in right hemisphere mu suppression for the mentalising task, though differences were borderline. Autistic traits (as measured by the Autism Spectrum Quotient (AQ) predicted right hemisphere mu suppression during the mentalising task. Left hemisphere mu suppression differed between groups only during the non-mentalising control task, and this difference was between two control groups (high vs low AQ), rather than between ASC and NT groups. Left hemispheric mu suppression during the mentalising condition predicted performance on the mentalising task. These findings are difficult to fit neatly with the BMH, which would predict group differences in mu suppression during both tasks, as reduced mu suppression during the mere viewing of actions has been considered supportive of the BMH in the studies described above (see Oberman et al., 2013). The BMH would also predict correlations between mu suppression, AQ scores and mentalising ability, given the characteristics of autism are argued to stem from atypicalities in the MNS. The stimuli for this experiment required the processing of goal-oriented actions, processing which the EP-M model argues is not abnormal in ASC: the minimal group differences could be explained this way. Alternatively, the group difference in mu suppression in the non-mentalising task could be explained by the STORM model, which argues that modulation of MNS activation is what is impaired in ASC; potentially top-down modulation failed to reduce MNS activity for the ASC group during this condition. However, this should lead to a lack of a task effect for the ASC group only, which is not reported by Cole et al. (in fact across all participants, there were no significant task effects, once only stimuli shown in both mentalising and non-mentalising condition were considered). Therefore, Cole et al.’s findings do not neatly fit with any of the three models, though the study concludes that ASC is associated with reduced right hemisphere MNS activity during mentalising.

Taken together, these EEG studies refute the BMH, as predicted differences in mu suppression were either not reported or are better explained under the alternative models, particularly the EP-M model. More recent studies that have examined mu suppression band differences at other sites and using other sub-bands have also produced findings fitting the STORM model. However, this model struggles to explain some of the findings brought about by studies using mentalising tasks: tasks in which we would expect social cues to modulate MNS activity.

### Evidence from TMS

TMS approaches to MNS research have typically utilised motor evoked potentials (MEPs). MEPs, movements stimulated by TMS over the motor cortex (e.g. arm flinches), are more pronounced during action observation, compared to a baseline condition. This has been suggested to represent pre-activation in the motor cortex during action observation; therefore, it is argued that differences in MEPs during action observation could provide a measure of the activity of the human MNS (Fadiga et al., 1995). Under the BMH, we would expect ASC participants to show no differences, or a reduced difference, in MEP responses during action observation versus a baseline condition, compared to controls. The EP-M hypothesis would suggest that this effect would depend on whether stimuli were goal-based or non-goal-based. STORM would predict effects would be dependent upon top-down modulation requirements. Compared to other methods, Hamilton (2013) found TMS to provide the most consistent results, in which ASC participants showed a lack of task modulation of MEPs when observing hand actions (Hamilton, 2013), a finding in keeping with the BMH.

Similar to Hamilton (2013), two studies that met the inclusion criteria were found to have collected TMS data: Enticott et al. (2013) and Cole et al. (2018). Cole et al. utilised the same paradigm as used in the EEG portion of their study (see above), whereas Enticott et al. asked participants to view videos of various hand actions: static hands, single hand actions or hands actions from two individuals interacting with one another.

No differences in MEPs were reported between ASC and NT participants in either investigation. This is intriguing as Cole et al. (2018) found significant group differences in MNS activity when using EEG (see above), despite incorporating the same participants. The conflicting reports between EEG and TMS measures suggest that these techniques may reflect activity from two different areas or cognitive processes. Indeed, it maybe that TMS-based MEPs reflect processes arising from the primary motor cortex, while EEG-derived mu suppression originates from the somatosensory cortex (Cheyne et al., 2003; Fadiga et al., 2005). Both areas have been shown to have mirror properties (Confalonieri et al., 2012); however, recent work with NT participants has suggested that the EEG measure of mu suppression mirrors the sensory aspects of an action, but not its motor components (Coll et al., 2015, 2017). Since group differences were only shown from EEG, these findings may suggest that purely the sensory aspects of the MNS are affected in ASC rather than the motor characteristics of the network.

Since Cole et al’.s stimuli were object-based and goal-directed (pushing a poker chip through a slot), the EP-M model would predict no MEP differences between ASC and NT participants, since these actions utilise the EP route. Enticott et al.’s (2013) stimuli involved interactive two-person hand actions; it is not clear whether such stimuli would be supported by the M or EP routes, but arguably one could conceive of these actions as having a goal. If so, a lack of group differences would be in keeping with the EP-M model.

The STORM model would predict that MEPs would be unaffected when viewing hand actions, as neither study was presented the task within a social context. However, deciding whether an actor is spiteful or clumsy could be argued to require social cues and therefore, ASC participants in the study by Cole et al. (2018) would be expected to show a lack of MEP modulation. The null effects are therefore contrary to STORM.

### Evidence from magnetic resonance imaging and diffusion tensor imaging studies

The brain regions typically considered to form the MNS usually include IFG and IPL. Magnetic resonance imaging (MRI) techniques allow for activity in these purported mirror neuron regions to be compared between ASC and NT groups. The BMH would predict global activity differences with the MNS, whereas the EP-M model would predict that differences lie within middle temporal regions, regions that support the M-route, and that activity differences would be specific to stimuli without objects. The STORM model predicts that differences in MNS activity, if any, are due to reduced top-down regulation. Therefore, differences in PFC activity should be present, and during tasks that require social modulation. In addition to differences in the activation of brain regions, diffusion tensor imaging (DTI) techniques allow for differences in the connectivity between regions belonging to the MNS to be investigated. Global connectivity differences would be in line with the BMH, whereas the EP-M model theorises that connectivity differences are specific to the M-pathway. Differences within top-down connections to the MNS would support the STORM account. Hamilton (2008, 2013) reported mixed evidence for the BMH in both reviews when reviewing the MRI literature. We identified 3 fMRI studies and 3 DTI studies.

Recent work using fMRI has reported significant differences in activity from MNS brain areas between the NT and ASC groups (Libero et al., 2014; Wadsworth et al., 2018). Both studies incorporated a mentalising task incorporating images of hands (see Table 1 for details). Higher MNS activity (in the IFG) was reported for the ASC participants in both studies. These results complement a recent meta-analysis of fMRI research, which noted hyperactivity in the IFG of ASC participants, when viewing different actions (Yang & Hofmann, 2016). Clearly, this direction is not the one typically predicted by the BMH: one could perhaps suggest that ASC participants have an inefficient MNS, which must work harder to equal the mentalising ability of NT individuals, but this post hoc explanation remains speculative (Libero et al. themselves suggest that their data show a relatively intact response of the MNS in ASC). Even so, hyperactivity localised within frontal and temporal areas would imply that inefficiencies reside within the M-pathway, supporting EP-M predictions, or within top-down connections to the MNS, in line with STORM predictions. Furthermore, in Wadsworth et al. (2018) the group differences in activity were only seen in conditions where participants viewed actions which lacked the use of an object. This could imply a specific abnormality in the M-pathway.

Reduced functional connectivity between the IFG and the MTG (i.e. the M-route) has been reported (Libero et al., 2014), up-holding EP-M predictions. However, the direction of these findings is inconsistent, and some have suggested that connectivity differences are not seen throughout the spectrum: Fishman et al. (2014) reported that the ASC participants with the greatest autistic symptoms demonstrated enhanced functional connectivity within the MNS (rather than reduced connectivity, as reported by Libero et al., 2014), and average connectivity within the MNS positively correlated with ASC symptomatology. However, which brain regions are incorporated into each network varies among reports: Fishman et al. (2014) included the IFG and MTG in the theory of mind network (TOMn) rather than the MNS, which may explain why only TOMn differences were reported in less severe ASC cases. When focusing on the individual brain regions rather than purported networks, participants who demonstrate strongest symptomatology showed enhanced functional connectivity between the IFG, MTG and mPFC. Fishman et al. (2014) also report enhanced connectivity between the TOMn and MNS: this could fit with the STORM model to suggest that unusual connections from the TOMn to the MNS lead to problems with top-down control, particularly affecting the M-route.

Given potential functional connectivity differences, investigating the white matter connections within the MNS may provide more insight into the abnormalities associated with ASC. Three studies using DTI, or more recent diffusion spectrum imaging (DSI), were identified: Schaer et al. (2013), Chien et al. (2015) and Fründt et al. (2018). Schaer et al. (2013) did not aim to specifically test the connectivity between mirror neuron regions: they do report reduced gyrification in the IFG (and IPL), and make the note that this area has been implicated in MN theories and that this reduced gyrification could indicate early developmental abnormalities, but this interpretation is made post hoc. Chien et al. (2015) and Fründt et al. (2018) failed to find group differences in MNS connectivity, findings which are problematic for the BMH and the EP-M model. A lack of group differences in MNS structure may suggest that atypicilaties lie outside of this system, as per the STORM model. Furthermore, Fründt et al. (2018) found no evidence for a fronto-temporal MNS tract in the ASC or NT group. This queries the existence of the M-pathway and the validity of the EP-M model. However, it should be noted that Hamilton’s (2008) review considers DTI evidence to support the existence of an M-pathway. Furthermore, other recent studies not specifically examining structures in the context of mirror neuron and related theories have reported connections between the IFG and MTG: Briggs et al. (2019) report that these regions are connected via the superior longitudinal fasciculus.

In summary, imaging techniques demonstrate that MNS activity and functional connectivity differ between ASC and NT individuals, although the direction of group differences is often contrary to expectations. In particular, the IFG is reported across different studies as having different activity, different functional connectivity and different structural properties, in ASC. This would suggest a dysfunction, as proposed by both the BMH and EP-M. However, DTI studies fail to show structural connectivity differences between participant groups within the MNS itself, and thus atypicalities may be the result of reduced top-down modulation, as argued by STORM.

### Evidence from near-infrared spectroscopy studies

Since near-infrared spectroscopy (NIRS) measures neural activity via oxy-haemoglobin levels, similar to fMRI, predictions from each model are identical to those made about MRI research. At the time of Hamilton’s (2013) review, no NIRS studies had been conducted. We identified one NIRS study investigating MNS function in ASC.

Mori et al. (2015) reported reduced IFG oxy-haemoglobin levels in ASC individuals compared to NT controls. This is intriguing given the hyperactivity reported in this region by Libero et al. (2014) and Wadsworth et al. (2018), using fMRI. Both methods essentially index blood-oxygen concentration levels and typically correlate in concurrent fMRI-fNIRS studies (e.g. see Cui et al., 2011). The participants used by Mori et al. (2015) had lower IQ scores than those of the fMRI studies (though IQ scores were still 70 and above, indicting no intellectual disability). It is unclear whether this could account for differences in levels of IFG activity; the recent meta-analysis by Yang and Hofmann (2016) of fMRI data also only included studies with participants with IQs within the normal range; thus, it appears that there are limited data to suggest whether lower IQs in autism pattern with underactivity of the IFG, as opposed to hyperactivity seen in autistic participants with higher IQs. Mori et al. (2015) also reported that activity in the IFG increased after the children with autism were taught to imitate facial expressions; however, the control group did not undergo this training, so relative increases in this area could not be examined.

Broadly, these findings support the BMH, in that the IFG, a key MNS area, is underactive, but training targeting behaviours that utilise the MNS can normalise the activity of the IFG. The involvement of the IFG, and its increase in activity after training of facial imitation – a form of imitation argued to rely particularly on the M-route – would also fit with the EP-M model.

### Evidence from post-mortem studies

For the data reviewed thus far, the resolution limitations of neuroimaging prevent discovering whether group differences in MNS activation are due to atypicality in mirror neurons themselves. Here post-mortem studies may be able to shed light on differences at the cellular level. The BMH would predict differences in the IFG and IPL, the EP-M would predict differences in the IFG and MTG and the STORM model would predict differences in frontal brain regions. Hamilton (2008, 2013) did not include post-mortem studies, but our search revealed one study of relevance: Jacot-Descombes et al. (2012).

This study measured neuronal differences within the IFG in eight post-mortem brains from ASC individuals and two NT brains. It was reported that ASC brains had reduced neuron size but equal neuronal frequency and layer volume within the IFG. Since all models would predict some form of neuronal difference within this area, affecting either the top-down pathways to the MNS (STORM model) or the efficiency of the M-pathway or entire MNS (EP-M model and BMH respectfully), such limited investigation cannot differentiate between the models in question. However, this reduction in neuron size could be responsible for the reduced connectivity between the IFG and PFC, shown in DTI studies. Neural tissue from individuals as young as 4 years old were incorporated, suggesting neural differences being present from early life. Therefore, such findings can add to evidence supporting BMH, EP-M and STORM accounts. However, there is no way of confirming whether these neurons had mirror properties.

### Evidence from NFT

NFT evidence with regard to the BMH argues that, assuming the BMH is valid, normalising MNS activity using NFT techniques should reduce ASC symptoms. In addition to its potential clinical uses, NFT could also provide an important experimental paradigm to acquire causal, rather than correlational, evidence regarding the role of the MNS in autistic symptomatology. NFT studies examining MNS function to date have relied on EEG measures of the MNS, in which children watch a film or play a game which is linked to EEG recordings of mu rhythms. The child must periodically decrease their mu rhythm (i.e. engage in MNS activity, as indexed by mu suppression) to continue the film or game while simultaneously ensuring that theta and beta activity (associated with distraction) remains below a threshold. If a deficit in the MNS is a cause of ASC symptomatology, as the BMH and EP-M models suggest, training based on MNS activity should reduce ASC symptoms (the EP-M model would specify that training M-route-based processes should be what supports a reduction in ASC symptoms). The STORM model would suggest that simply normalising activity will not help reduce autistic symptoms, rather training ought to consider the appropriate modulation of mirroring processes.

Studies into the effectiveness of NFT (Datko et al., 2018; Pineda et al., 2008, 2014) claim to show such findings. Furthermore, by combining NFT with fMRI, Datko et al. (2018) reported that NFT increased activity within the MNS in ASC participants.

Intriguingly, NFT may have disadvantageous effects on NT participants, including reduced social communication scores (Pineda et al., 2014) and reduced MNS activity measured by fMRI (Datko et al., 2018). It is possible that the disadvantageous effects of NFT on NT individuals result from the MNS becoming hyper-activate during NFT, comparable to the hyper-activity seen in ASC participants in some fMRI studies (e.g. Libero et al., 2014; Wadsworth et al., 2018). However, such findings do not rule out that changes in MNS activity may be due to punishing attentional laps, and therefore improving top-down control to the MNS, as expressed by the STORM model.

The NFT literature at present faces several methodological issues. Neither Pineda et al. (2014) or Datko et al. (2018) used a placebo NFT condition with ASC participants, and both studies relied on parental reports to measure behavioural improvements. This is clearly not a blind test, opening the possibility that parental expectations about the effectiveness of NFT confounded the improvements seen in the ASC group. This is especially worrisome given the stated motivations for parents to participate in the study: ‘Parents and children with ASD were primarily motivated by the expectation that the training would produce differences in behaviour’ (Pineda et al., 2014, p. 3).

Only Pineda et al. (2008) incorporated an ASC placebo group and a double-blind protocol. The study reported that NFT did improve behavioural measures only in the experimental group; however, these improvements were limited to attention and not ASC-specific symptoms. This increases the likelihood that improvements in ASC symptomatology are linked to increased prefrontal control (Kane & Engle, 2002), caused by punishing attentional shifts. Therefore, results are perhaps better explained by the STORM model or possibly a general attentional model (e.g. Chun et al., 2011) rather than the BMH. Furthermore, these paradigms may have failed to improve other symptoms (e.g. deficits in social interaction) because children were taught to modulate via explicit input (e.g. the film stopping and starting). Arguably, this does not represent the cues used, according to the STORM model, to modulate mirroring activity usually: perhaps if social cues were used, as suggested by the STORM model, improvements in social and communication may be seen.

To summarise, the NFT literature contains methodological flaws, and the most prominent study in the literature only shows improvements in attention, which could be better explained by the STORM model.

## Discussion

The BMH has been a highly influential (if controversial) theory regarding autism, and it is evident that this theory and the counter-hypotheses that came after it continue to inform neuroscientific research in autism. Consistent with previous reviews, this review found a significant lack of evidence for the BMH. The EP-M model received some support from EEG, TMS and fMRI evidence. Some DTI evidence included in this review cast some doubt over the validity of this model, given that lack of a neuroanatomical connection between the brain region comprising the M-pathway, although such a pathway has been identified in non-MNS related research. The STORM model also received some support, particularly from EEG/NFT data, and (unlike the EP-M model) is not dependent upon the existence of an M-pathway. As noted by Hamilton (2008), these two models are not necessarily mutually exclusive, and selective problems with M-route-related processes could be caused by problems with top-down modulation. Indeed, this is perhaps the best way to reconcile the evidence as both the majority of STORM and EP-M predictions are supported.

Future researchers should consider investigating the nature of top-down modulation in ASC, possibly implemented by the PFC, as opposed to just examining the MNS itself. The PFC has been implicated in allocating the contextual goal behind a task (Blakemore & Decety, 2001; Erlhagen et al., 2006; Mante et al., 2013) and has been linked to action inhibition (Ridderinkhof et al., 2004; Swick et al., 2008) in NT participants. However, the exact nature of the top-down regulation required remains unclear. A possible candidate could be a process referred to as self-other control, in which the activity of neural representations referring to self-executed actions and representations stimulated by the actions of others are modulated depending on the social situation (de Guzman et al., 2016; Sowden & Shah, 2014). For example, individuals must reduce their own internal state and enhance neural representations externally driven by another’s emotional state when empathising with an individual. In contrast, one must inhibit motor representations stimulated by observed actions and increase internally driven motor representations to prevent imitation of another person’s actions. Training self-other control in the motor domain has been shown to improve empathy scores (de Guzman et al., 2016) and perspective taking (Santiesteban et al., 2012), the latter being a specific effect to imitation-inhibition training, as general inhibition training had no effect. This suggests that self-other control could be a general mechanism behind a variety of social cognitive abilities (e.g. imitation, theory of mind, empathy, etc.). Since self-other control has been consistently linked to mPFC and temporoparietal junction (TPJ) activity (Brass et al., 2001, 2005, 2009; Liepelt et al., 2016; Sowden & Shah, 2014), the reduced self-other control seen in ASC (Sowden & Shah, 2014; Spengler et al., 2010) mirrors the lack of top-down regulation proposed by the STORM model.

A strength of the STORM account is that it is not dependent upon the existence of the MNS itself. Even though Hamilton incorporated the MNS within the model, the model is mainly discussed in terms of a basic visual-motor mapping system, consisting of the inferior parietal cortex, premotor cortex and primary motor cortex. Therefore, evidence which contradicts the existence of a human MNS (e.g. Fründt et al., 2018; Lingnau et al., 2009) would not pose an issue to the model’s validity, as the model focuses on the top-down regulation of a visual-motor system via social cues, rather than the nature of how visual-motor mapping is achieved. This is not the case for the BMH or EP-M model, which both specifically require the existence of the MNS in humans.

It is possible that discrepant findings may be explained by factors such as participants’ age and IQ levels. With regard to how age and gender bear upon our analysis of these studies, we see no particular pattern with age: results that refute the BMH occur across studies with different ages of participants and different proportions of males and females. For IQ, at least for the studies that included details regarding the IQ of their samples, it appears that these studies largely only included participants without intellectual disability (i.e. IQ > 70). Thus, it must be borne in mind that the results reported in these studies, and thus the conclusions of our review, are restricted to autistic people without intellectual disabilities.

In conclusion, this review extends the current ASC literature by specifically contrasting the BMH, EP-M and STORM model against each other, based on recent neuroscientific evidence. Evidence contradicts predictions made by the BMH and favours an integrated account of the EP-M and STORM models. Future investigations should focus on top-down modulation, rather than simple MNS dysfunctions in ASC. Such investigations should use mentalising or facial imitation tasks, which may be more sensitive to detecting specific processes important to the STORM model (e.g. regulation via social cues) than the often used hand observation tasks. Furthermore, we suggest shifting away from looking for dysfunction in isolated MNS areas, in favour of examining how networks regulate multiple systems, which contribute to social processes in both NT and ASC populations.
